# Self-Paced Walking within a Diverse Topographical Environment Elicits an Appropriate Training Stimulus for Cardiac Rehabilitation Patients

**DOI:** 10.1155/2012/140871

**Published:** 2012-07-09

**Authors:** James Faulkner, Johannes Gerhard, Lee Stoner, Danielle Lambrick

**Affiliations:** ^1^School of Sport and Exercise, Massey University, Private Bag 756, Wellington 6140, New Zealand; ^2^Institute for Sport Sciences, Julius-Maximilians-University, 97070 Wuerzburg, Germany; ^3^Institute of Food, Nutrition and Human Health, Massey University, Wellington, New Zealand

## Abstract

*Purpose*. To assess the effect of a self-paced walking intervention within a topographically varied outdoor environment on physiological and perceptual markers in cardiac rehabilitation (CR) patients. *Methods*. Sixteen phase II CR patients completed twelve self-paced one-mile walking sessions over a four-week period within a community-based CR programme. Walking velocity, heart rate (HR), and ratings of perceived exertion (RPE) were reported at eight stages throughout the self-paced walks. *Results*. The study showed a significant increase in walking velocity from week 1 (*~*4.5 km/h) to week 4 (*~*5.1 km/h) of the self-paced walking programme (*P* < .05). A significantly higher HR was also observed in week 4 (111 ± 13 b*·*min^−1^; *~*69% of maximal HR) compared to week 1 (106 ± 14 b*·*min^−1^; *~*65% of maximal HR, *P* < .001). There were no changes in the average RPE across the course of the 4-week self-paced walking programme (*P* > .05). *Conclusion*. A self-paced walking programme may elicit an appropriate training stimulus for CR patients when exercising within a diverse topographical environment. Participants completed a one-mile walk within a shorter period of time and at a higher physiological intensity than that elicited at the onset of the programme, despite no observed changes in participants' subjective perception of exertion.

## 1. Introduction

Cardiac rehabilitation (CR) programmes promote active lifestyles through the adherence to physical training and compliance to healthy behaviours [1]. Both cycling and walking are employed during CR to reduce cardiac risk factors and elicit improvements in peak exercise capacity and health-related quality of life [2, 3]. Of these two exercise modes, walking may be considered a more accessible, familiar, and habitual form of exercise for the average individual [4]. 

Prescriptive exercise is widely assumed to be the cornerstone of an effective CR programme [5]. There is however evidence that some individuals struggle to reproduce or accurately recall their exercise intensity within a prescribed training environment [6–8]. High-intensity exercise may also be dangerous and induce complications for CR patients [5]. If the enjoyment associated with an exercise programme is reduced, or the perception of pain is elevated following prescribed exercise of a vigorous nature, an individual's long-term exercise adherence may be affected [5]. 

Heart rate (HR) and the ratings of perceived exertion (RPE) are often used to monitor and regulate exercise intensity [9]. Despite this, approximately 85% of individuals use an inherent sense of effort to adjust and control exercise intensity [5]. For CR patients, self-paced exercise has recently been advocated as an alternative approach to improve fitness and health, and to enable sustained, lifelong behavioural change [5]. By augmenting the feelings of pleasure and minimizing the perception of displeasure, self-selected exercise, which is often undertaken within recommended exercise intensities [10], has been associated with enhanced adherence to exercise programmes [5, 11].

During a 3-week aerobic exercise programme, CR patients have been shown to elicit heart rate intensities at or above a recommended moderate training intensity (49–69% of heart rate reserve) when self-selecting their exercise intensity [12]. Similar findings were observed with 142 phases II and III CR patients during a one-mile brisk walk on a flat surface [13]. In this study, participants exercised at 79–85% of age predicted maximal heart rate (HR_max_), a self-selected intensity which was similar to American Heart Association (50–70% HR_max_) and the American College of Sports Medicine (>70% HR_max_) recommendations [9, 14]. As the terminal RPE was between 12 and 14, which is within the safe exercise recommendations of the ACSM, the authors concluded that brisk walking is an appropriate means for CR patients to achieve cardio-respiratory benefit. However, a significant proportion of studies have only assessed the utility of walking on a treadmill or on a flat surface [13, 15, 16]. To ensure that an exercise programme has sustained and pragmatic implications for improving an individual's ability to complete and adhere to activities of daily living, it is necessary to assess the effect of self-paced walking in an environment that complements everyday life [1, 17, 18]. 

The purpose of this study was to assess the effect of self-paced walking in a diverse topographical environment on physiological and perceptual markers in CR patients. We hypothesised that short-term (4 weeks) self-paced exercise would elicit an appropriate training stimulus for CR patients that would lead to improvements in cardio-respiratory fitness.

## 2. Methods

### 2.1. Subjects

Sixteen Phase II CR patients (12 male, 4 female; mean ± SD, 64.5 ± 8.9 y; 1.72 ± 0.07 m; 88.6 ± 13.4 kg; 30.2 ± 3.9 kg/m^2^), participated in the study. All patients were referred from local hospitals following a major cardiac event (myocardial infarction, coronary artery bypass surgery, etc.) to a community-based CR programme. Patients were not being treated with antidepressive medications, and dosages of all other medications were stable for at least 4 weeks prior to entry to the study. Participants provided written informed consent and had no prior experience of perceptual scaling, such as using the Borg 6–20 Ratings of Perceived Exertion (RPE) [19]. Research was approved by the New Zealand Central Regional Health and Disability Ethics Committee and was conducted to conform with the Code of Ethics of the World Medical Association (Declaration of Helsinki).

### 2.2. Procedures

**Figure 1:**
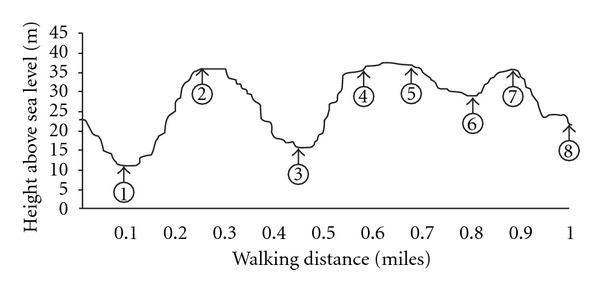
Topography of one-mile walk. Numbers 1 to 8 relate to the location (Stages) where data was collected.

Participants completed a peak/symptom-limited exercise ECG stress test using a modified Bruce protocol, and an ACSM health risk assessment prior to study participation. Participants then completed twelve self-paced, one-mile walking sessions on a predetermined walking route in an outside environment. The walking route was of varying topography (incline, decline) and consisted of eight distinct stages (Figure 1). The maximal change in course elevation was 25 m. Walking sessions commenced at 7.00 am to limit inter- and intraindividual circadian rhythm variation. Participants completed three sessions each week, with a 48-hour recovery period between sessions. Participants were familiarized with the walking route and study equipment prior to the initial exercise session. Study equipment included a HR monitor and watch (Polar Electro T31, Kempele, Finland), the Borg 6–20 RPE scale, and a permanent marker pen. Participant's HR, RPE and walking velocity were monitored throughout each exercise session. Investigators were located at predetermined locations along the walking route to ensure that participants did not deviate from the course. Systolic (SBP) and diastolic (DBP) blood pressure was measured using an Aneroid Sphygmomanometer (Accoson Works, London, UK) prior to, immediately following and five minutes after each walking session.

### 2.3. Measures

#### 2.3.1. Heart Rate and Walking Velocity

Heart rate was measured prior to-, during and following each session. At the start of each walk, participants started the “timer” on their HR watch. Heart rate was averaged every 5 seconds for the duration of the walk. The peak HR value obtained at the end of each walking stage was used to represent the HR response from that stage of exercise. The HR watch was also used to electronically store participants split time at the completion of each of the eight stages of the walk. By ascertaining the time to complete a specific stage, and by knowing the distance of that stage, walking velocity (km/h) was subsequently calculated.

#### 2.3.2. Ratings of Perceived Exertion (RPE)

Participants' “overall” feelings of exertion were reported at the completion of each exercise stage. Participants were perceptually anchored to the Borg 6–20 scale prior to each test (i.e., RPE 9, 13 and 19) after receiving standardised written and verbal instructions. Participants completed the walk holding a Borg 6–20 RPE scale and a permanent marker pen. Participants noted their chosen perception of exertion directly on the scale at the end of each stage of the test.

### 2.4. Data Analysis

Data was pooled into four, one week blocks (three walking sessions/week). A one-way repeated-measures ANOVA was used to assess whether there was a significant change in the average time to complete the one-mile walk. Thereafter, two-way repeated-measures ANOVAs, Week [1 to 4] × Stage [1 to 8] were used to analyse participants' HR, % HR_max_, RPE, and walking velocity from the walking programme. A repeated-measures contrast was used to locate consecutive main effects for Weeks and Stages. Post hoc pairwise comparisons were used to compare values from week 3 and week 4 with week 1 for each of the above markers. Where significant interactions (Week × Stage) were observed, Tukeys HSD criterion was used to identify the location of the statistical finding. Alpha was set at  .05 and adjusted accordingly. All data was analysed using SPSS version 18.

## 3. Results

A one-way ANOVA revealed a significant reduction in the average walking duration across Weeks (*F*_(1.9,28.4)_ = 72.4, *P* <  .001). Post hoc analysis demonstrated a significant decrease in walking duration between each consecutive week (Table 1).

A series of two-way repeated-measures ANOVA revealed a main effect across Weeks for walking velocity (*F*_(1.5,  22.1)_ = 57.9, *P* < .001) and HR (*F*_(3,45)_ = 14.3, *P* < .001). Post hoc analysis revealed a significant increase in walking velocity between consecutive weeks (all *P* <  .05; Table 1). A significant change in HR was only observed between week 2 and 3 (*P* <  .001), although HR in week 3 and 4 were both significantly higher than the average HR observed in week 1 (both *P* < .05; Table 1). Similar findings were observed when HR was expressed as % HR_max_. There were no differences in the average RPE across all Weeks (*P* > .05).

**Figure 2:**
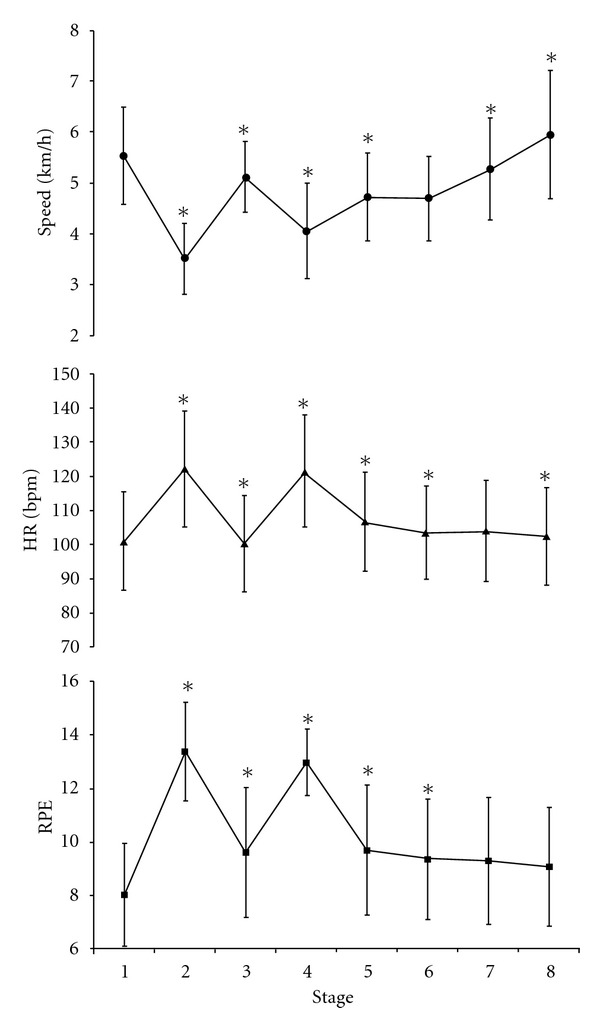
Means (±SD) of walking velocity, HR and RPE across the eight stages of the self-paced walk. *Significant change from antecedent stage (*P* < .05).

A significant main effect for Stage was observed for walking velocity, HR, and RPE (all *P* < .05; Figure 2). Consecutive changes were observed for all but one Stage for walking velocity (Stage 5 to 6; *P* > .05) and HR (Stage 6 to 7; *P* > .05), and all but two stages for RPE (Stage 6 to 8; *P* > .05).

A significant Week by Stage interaction was only observed for walking velocity (*F*_(3.5,53.1)_ = 3.33, *P* < .001). Post hoc analysis using Tukey's HSD criterion indicated that the rate of change in walking velocity between stage 6 and 7, and stage 7 and 8 was significantly less for Week 2 and Week 1 than all other weeks, respectively (*P* < .01). The average walking velocity in week 4 was (on average) 0.56, 0.43, 0.59, 0.64, 0.38, 0.63, 0.79, and 1.20 km/h faster than Week 1 for Stages 1 to 8, respectively.

## 4. Discussion

This study assessed the efficacy of self-paced walking on physiological and perceptual markers in CR patients. The designated course elevation mimicked the natural variation in topography associated with many community and local environments. This study extends the encouraging findings for the use of walking as a means of eliciting an appropriate training stimulus for CR patients [2, 13, 19].

In this study, participants selected an appropriate walking velocity without prior task familiarisation. Participants exercised at ~64% HR_max_ within the first week of exercise which is within recommended guidelines (50–70% HR_max_) to elicit cardio-respiratory benefit for CR patients [14]. However, following 3 weeks of self-selected exercise, a significant increase in HR was observed (~67% HR_max_). These findings are comparable to previous studies (60–70% HR_max_) that have implemented self-paced exercise with healthy [20] and obese [21] subjects. However, when subjects are requested to walk at a “brisk” pace, research has revealed a higher exercise intensity (~79–85% HR_max_) than that observed in the present study [13, 21]. It may be speculated that discrepancies in participant instructions (i.e., self-paced, preferred or brisk walk) may contribute to the observed variation in exercise intensity. 

As a result of the diverse topography, participants were physically challenged on two occasions for a period of ~30 s to 1 min (Stage 2 & 4). The 18 m change in course elevation stimulated HR to peak at ~75% HR_max_. High-intensity interval training has recently been advocated with coronary artery disease patients [22] and heart failure patients [23, 24]. As such, continuous, low-moderate exercise interspersed with short periods of high-intensity exercise may be of advantage, as it may help to augment the feeling of pleasure during lower intensity exercise whilst attenuating perceptions of displeasure that are associated with prolonged high-intensity exercise [11, 25, 26].

It is plausible that the observed fluctuations in exercise intensity are resultant upon participants consciously pacing themselves throughout the walking sessions. Four broad pacing strategies have been described with athletes: “all out” (fast start), “slow start,” “even pace,” and “variable” strategies. A 12% increase in the average walking velocity in the current study was observed between week 1 and week 4 (~4.5 cf. ~5.1 km/h, resp.), despite no differences being observed in the pacing strategy adopted. Participants walked between 0.38 and 1.20 km/h faster in week 4 at various stages to the one-mile route than observed during week 1. Due to course topography, it is unsurprising that participants utilised a “variable” pacing strategy. Typically, walking velocity increased when participants walked downhill or on the flat. However, a considerable increase in walking velocity was observed in the final two stages of the walk, despite an increase in course elevation between the sixth and seventh stage. It has been suggested that when participants can conceptualise the completion of an exercise bout, an increase in exercise intensity may occur to maximize exercise performance [27]. Although this has previously been observed during cycling and running, this is the first study to demonstrate such changes during walking. 

The 11.6% improvement in performance, as demonstrated by a decrease in exercise duration from 21.7 min (week 1) to 19.2 min (week 4), is comparable to findings observed during longer duration exercise interventions [28]. The first notable change in walking duration occurred within the first two weeks of the intervention. Yet, as increases in HR were only observed in week 3, improvements in exercise performance prior to this may be attributed to task familiarisation, and both intra- and intermuscular coordination [29]. As self-selected exercise may provide participants with a greater sense of control and elevated levels of interest, enjoyment, and perceived autonomy [5], this may provide the foundation for an elevated physiological drive. The overall improvement in performance was comparable to the 14% increase in average power output that was observed during a 3-week cycle-based programme for cardiac patients [12]. Despite the short duration of the walking programme, participants' improvement in exercise performance is comparable to those that have utilised a longer exercise intervention [28, 30]. 

Although participants completed the one-mile walk with a higher average HR and walking velocity after 4 weeks of exercise, there were no changes in participants' effort perception. Participants reported a mean RPE of 10 throughout the exercise programme, which was similar to that observed with non-CR participants when requested to exercise at a preferred or self-selected intensity [5, 19, 20]. However, higher RPEs have been reported when participants have been requested to walk at a “brisk” pace outside [13, 20] or when asked to exercise at a “preferred” intensity on a treadmill [31]. Due to the diverse topography of the intervention, perceptions of exertion mirrored the changes in course elevation, with peak RPEs reported at stage 2 and 4 (~RPE 13). This “somewhat hard” perception of exertion is generally considered to be a suitable and beneficial training stimulus for cardio-respiratory adaptations [9]. Due to the differences in perceptual and physiological responses elicited during self-selected and prescribed exercise (i.e., brisk walking), future research should consider the effect of participant instructions on the training stimulus.

## 5. Conclusion

This study has demonstrated that self-paced walking within a diverse topographical environment may elicit an appropriate training stimulus for CR patients. Following a 4 week (12 session) intervention, participants completed a one-mile walk faster and at a higher physiological intensity than that elicited at the onset of the programme. This was apparent despite no changes in participants' subjective perception of exertion. Self-paced walking within a varied outdoor topography is therefore recommended as a rehabilitation strategy for cardiac patients.

## Figures and Tables

**Table 1:** Mean (±SD) performance, physiological, and perceptual responses from weeks 1 to 4.

	Week 1	Week 2	Week 3	Week 4
Time (min)	21.4 ± 3.1	20.4 ± 3.1^∗†^	19.6 ± 3.2^∗†^	18.8 ± 3.0^∗†^
Walking velocity (km/h)	4.6 ± 0.6	4.8 ± 0.7^∗†^	5.0 ± 0.7^∗†^	5.3 ± 0.7^∗†^
HR (bpm)	106 ± 14	107 ± 14	111 ± 16.0^∗†^	111 ± 13.0^†^
% HR_max⁡_	64.7 ± 8.8	65.6 ± 9.0	68.0 ± 10.1^∗†^	68.2 ± 8.8^†^
RPE	9.9 ± 1.6	10.2 ± 1.6	10.1 ± 1.8	10.1 ± 1.9

*Significant change from antecedent week (*P* < .05). ^†^Significant change from Week 1 (*P* < .05).
